# Another Year of Record Heat for the Oceans

**DOI:** 10.1007/s00376-023-2385-2

**Published:** 2023-01-11

**Authors:** Lijing Cheng, John Abraham, Kevin E. Trenberth, John Fasullo, Tim Boyer, Michael E. Mann, Jiang Zhu, Fan Wang, Ricardo Locarnini, Yuanlong Li, Bin Zhang, Fujiang Yu, Liying Wan, Xingrong Chen, Licheng Feng, Xiangzhou Song, Yulong Liu, Franco Reseghetti, Simona Simoncelli, Viktor Gouretski, Gengxin Chen, Alexey Mishonov, Jim Reagan, Guancheng Li

**Affiliations:** 1 International Center for Climate and Environment Sciences, Institute of Atmospheric Physics, Chinese Academy of Sciences, Beijing, 100029 China; 2 Center for Ocean Mega-Science, Chinese Academy of Sciences, Qingdao, 266071 China; 3 University of St. Thomas, School of Engineering, Minnesota, 55105 USA; 4 National Center for Atmospheric Research, Boulder, Colorado 80307 USA; 5 University of Auckland, Auckland, New Zealand; 6 National Oceanic and Atmospheric Administration, National Centers for Environmental Information, Silver Spring, Maryland 20910 USA; 7 Department of Earth and Environmental Science, University of Pennsylvania, Philadelphia, Pennsylvania 19104 USA; 8 Institute of Oceanology, Chinese Academy of Sciences, Qingdao, 266071 China; 9 National Marine Environmental Forecasting Center, Ministry of Natural Resources of China, Beijing, 100081 China; 10 College of Oceanography, Hohai University, Nanjing, 210098 China; 11 National Marine Data and Information Service, Tianjin, 300171 China; 12 Italian National Agency for New Technologies, Energy and Sustainable Economic Development, S. Teresa Research Center, Lerici, 19032 Italy; 13 Istituto Nazionale di Geofisica e Vulcanologia, Sede di Bologna, Bologna, 40128 Italy; 14 South China Sea Institute of Oceanology, Chinese Academy of Sciences, Guangzhou, 510301 China; 15 ESSIC/CISESS-MD, University of Maryland, College Park, MD, College Park, Maryland 20740 USA; 16 Eco-Environmental Monitoring and Research Center, Pearl River Valley and South China Sea Ecology and Environment Administration, Ministry of Ecology and Environment, PRC, Guangzhou, 510611 China

**Keywords:** ocean heat content, salinity, stratification, global warming, climate, 海洋热含量, 盐度, 层结, 全球变暖, 气候

## Abstract

Changes in ocean heat content (OHC), salinity, and stratification provide critical indicators for changes in Earth’s energy and water cycles. These cycles have been profoundly altered due to the emission of greenhouse gasses and other anthropogenic substances by human activities, driving pervasive changes in Earth’s climate system. In 2022, the world’s oceans, as given by OHC, were again the hottest in the historical record and exceeded the previous 2021 record maximum. According to IAP/CAS data, the 0–2000 m OHC in 2022 exceeded that of 2021 by 10.9 ± 8.3 ZJ (1 Zetta Joules = 10^21^ Joules); and according to NCEI/NOAA data, by 9.1 ± 8.7 ZJ. Among seven regions, four basins (the North Pacific, North Atlantic, the Mediterranean Sea, and southern oceans) recorded their highest OHC since the 1950s. The salinity-contrast index, a quantification of the “salty gets saltier—fresh gets fresher” pattern, also reached its highest level on record in 2022, implying continued amplification of the global hydrological cycle. Regional OHC and salinity changes in 2022 were dominated by a strong La Niña event. Global upper-ocean stratification continued its increasing trend and was among the top seven in 2022.

## Abstract (zh)

由于人类活动排放温室气体, 全球能量和水循环已经发生了显著的变化, 驱动了气候系统的一系列变异. 海洋热含量、 盐度和层结变化是地球系统能量和水循环的重要指针. 2022 年, 全球海洋上层 2000 米热含量再破记录, 海洋成为有现代记录以来最热的一年. 据中国科学院大气物理研究所的测算, 2022 年 0–2000 米海洋热含量超过 2021 年 10.9 ± 8.3 泽塔焦耳 (1 泽塔焦耳= 10^21^焦耳). 与之一致, 美国国家海洋和大气管理局国家环境信息中心的测算为 9.1 ± 8.7 泽塔焦耳. 在所研究的 7 个海盆中, 北太平洋、 北大西洋、 地中海、 南大洋这 4 个海盆的 2022 年度热含量均创下了自上世纪 50 年代以来的新记录. 此外, 定量化测算海洋盐度 “咸变咸, 淡变淡” 变化趋势的 “盐度差指数” 也在 2022 年达到过去半世纪以来的最高值, 反映了全球水循环在不断加速. 在区域尺度, 海洋热含量和盐度变化显示出较强的拉尼娜事件的影响. 最后, 全球上层 2000 米海洋层结也持续加强, 2022 年全球海洋层结处于有现代记录以来的第 7 高位.

## 1. Introduction

Driven by anthropogenic greenhouse gas emissions, there is an energy imbalance in the Earth's climate system (Trenberth et al., 2009, 2014; Hansen et al., 2011; von Schuckmann et al., 2016, 2020; Cheng et al., 2022a). More than 90% of the excess heat accumulated in the climate system is deposited in the world's oceans (Rhein et al., 2013; Johnson et al., 2018; Cheng et al., 2019). The ocean heat content (OHC) influences ocean–atmosphere interactions by providing thermal inertia to sea surface temperatures and thus exerts considerable control over the world's weather (Cheng et al., 2022a). Rising ocean temperatures bolster the energy exchanges from ocean to atmosphere, increase the quantity of atmospheric moisture, and change the patterns of precipitation and temperature globally (Cheng et al., 2022a). As OHC is less impacted by internal climate variability [e.g., El Niño–Southern Oscillation (ENSO)], it is a particularly robust metric of global climate change (Cheng et al., 2017b).

Salinity is another key physical property of seawater and, together with temperature, it determines the water density, which is a vital driver of ocean circulation. The changes in ocean salinity reflect the global exchanges of surface freshwater. Evaporation refers to the transfer of freshwater from a water body to the atmosphere, leaving behind liquid water that is higher in salinity. On the other hand, precipitation injects freshwater into otherwise saline water, resulting in freshening. The salinity anomalies associated with surface freshwater exchanges are then dispersed in the ocean through ocean circulation and mixing (Schmitt, 1995; Durack, 2015; Yu et al., 2020). Consequently, salinity changes integrate effects over broad areas and provide an excellent indicator of water cycle change and variability. To quantify these changes, the Salinity Contrast (SC) index is employed, defined as the difference between the salinity averaged over climatologically high-salinity and low-salinity regions, and provides a simple but powerful means of diagnosing the observed salinity pattern changes (Cheng et al., 2020).

Temperature and salinity changes alter the ocean density and lead to changes in vertical stratification. The stratified configuration of the ocean can act as a barrier to water mixing, which impacts the efficiency of vertical exchanges of heat, carbon, oxygen, and other constituents (Li et al., 2020a). Thus, stratification is a central element of Earth's climate system, and understanding its changes in conjunction with global warming has important scientific, societal, and ecological consequences.

Accordingly, the OHC, salinity, and stratification are important as metrics for quantifying climate change, as well as through the influence of the oceans on weather and society (Abraham et al., 2022). This paper provides an update on the OHC in 2022 using two different data products—that of the Institute of Atmospheric Physics (IAP) at the Chinese Academy of Sciences (CAS) (Cheng et al., 2017a, 2020; Li et al., 2020a), and that of the National Centers for Environmental Information (NCEI) at the National Oceanic and Atmospheric Administration (NOAA) (Levitus et al., 2012). The SC index and stratification were updated using IAP data. These groups correct systematic errors in data, and both use mapping methods to relate discrete measurements into a continuous picture of the world's oceans.

## 2. Data and methods

Data are obtained from *in situ* measurements made available through the World Ocean Database. The instruments used to collect data include expendable bathythermographs (XBTs), Argo profiles (Argo, 2022), conductivity/temperature/depth instruments, mechanical bathythermographs (MBTs), bottles, moorings, and gliders (Boyer et al., 2018). Each of these instruments has an associated accuracy that may vary over time or with geographical location (Abraham et al., 2013; Cowley et al., 2021). Efforts have been made by the scientific community to improve the accuracy of these instruments. Although the source data are the same for the two groups, the gap-filling approach and many other data processing techniques are different. As an example, XBT biases have been corrected for IAP by Cheng et al. (2014) and for NCEI by Levitus et al. (2009). The newly available corrections for bottle and MBT data (Gouretski and Cheng, 2020; Gouretski et al., 2022) have not been incorporated; instead, MBT biases have been corrected for NCEI by Levitus et al. (2009) and for IAP by Ishii and Kimoto (2009). Besides, a reanalysis data from the Copernicus Marine Service (CMS) is used for the Mediterranean Sea (CMS-MEDREA, Escudier et at., 2020 and Nigam et al., 2021). The CMS-MEDREA assimilated XBT, CTD, Argo profiles, integrating data from CMS and SeaDataNet (https://www.seadatanet.org/) and, CMS satellite along track sea level anomaly (Escudier et al., 2021).

The 0–2000 m SC index is calculated as in Cheng et al. (2020) for each month ($t$) over the 3D ($x$, $y$, $z$) ocean salinity field:

$$\mathrm{SC}(t) = \frac{\iiint_{V_{\mathrm{high}}} S\,(x,y,z,t)\,\mathrm{d}V}{\iiint_{V_{\mathrm{high}}} \mathrm{d}V} - \frac{\iiint_{V_{\mathrm{low}}} S\,(x,y,z,t)\,\mathrm{d}V}{\iiint_{V_{\mathrm{low}}} \mathrm{d}V}\,, \quad (1)$$

where ($x$, $y$, $z$) are the three dimensions of latitude, longitude, and depth; $V_{\mathrm{high}}$ is the salinity averaged over high-salinity regions ($V_{\mathrm{high}}$) where salinity is higher than the climatological global median $S_{\mathrm{clim}}$; and $V_{\mathrm{low}}$ is the salinity averaged over low-salinity regions ($V_{\mathrm{low}}$) where salinity is lower than the climatological global median $S_{\mathrm{clim}}$. $S_{\mathrm{clim}}$, $V_{\mathrm{high}}$ and $V_{\mathrm{low}}$ are all determined on the basis of the climatological salinity field during 1960–2017. All data collected in the World Ocean Database (Boyer et al., 2018) are used to calculate the SC index, including real-time Argo observations.

Ocean stratification is calculated as in Li et al. (2020a), computed as the squared buoyancy frequency:

$$N^2 = gE = g\left[-\left(\frac{1}{\rho}\right)\left(\frac{\partial \sigma_n}{\partial z}\right)\right], \quad (2)$$

where $\rho$, $\sigma_n$ and $g$ denote the sea water density, local potential density anomaly, and gravitational acceleration, respectively; and $N$, the Brunt–Väisälä frequency, represents the intrinsic frequency of internal waves.

We present the most up-to-date information from both IAP and NCEI for 2022, incorporating the most recent data quality processing and mapping techniques. Those improvements result in modest changes in previous estimates, and we also provide recalculated 2019–21 OHC values using the most recent analysis from IAP and NCEI. The upper 2000 m ocean volume is analyzed for all these parameters because of the data availability and the capability of the current data processing techniques. For instance, previous studies suggest a reliable estimate of the upper 2000 m OHC, SC index, and stratification since the late 1950s (Levitus et al., 2012; Cheng et al., 2017a, 2020; Li et al., 2020a).

## 3. Global ocean changes in OHC, salinity, and stratification

Figure 1 shows the changes to the global upper 2000 m OHC since 1958. Regardless of the processing techniques, there is an unequivocal ocean warming trend in recent decades. The NCEI three-month OHC estimate has a

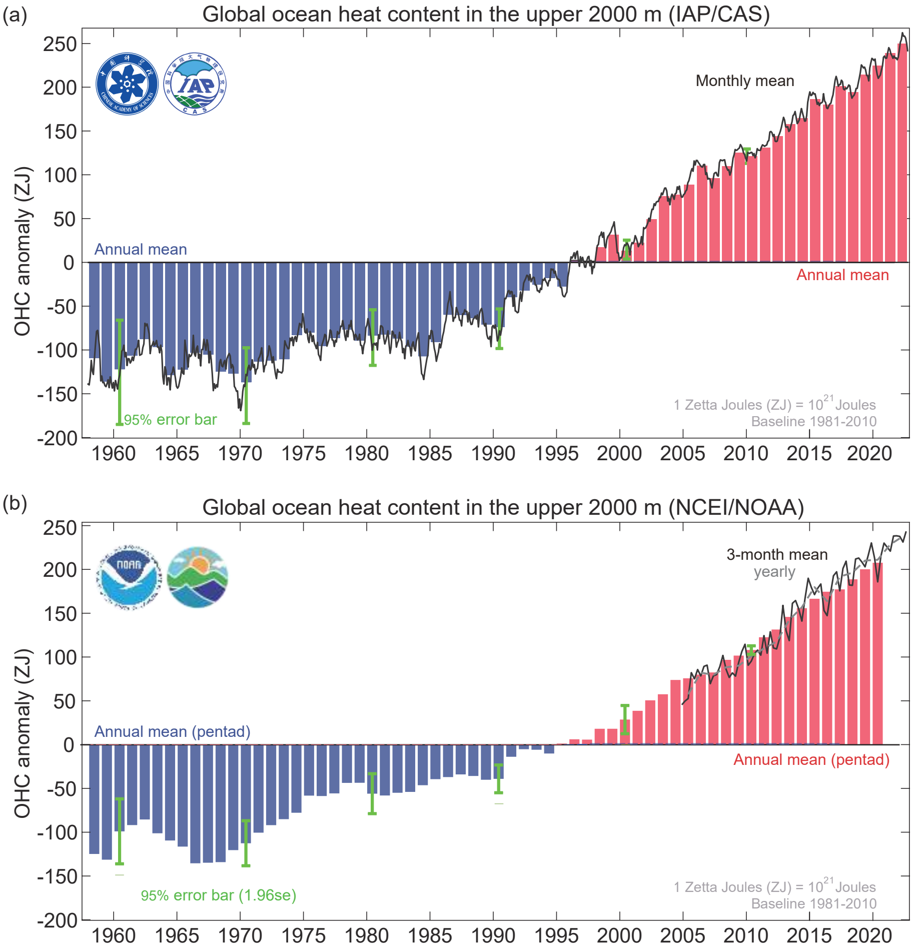

**Fig. 1.** Global upper 2000 m OHC from 1958 through 2022 according to (a) IAP/CAS and (b) NCEI/NOAA data. 1 ZJ = $10^{21}$ Joules. The line shows (a) monthly and (b) seasonal values, and the histogram presents (a) annual and (b) pentad anomalies relative to a 1981–2010 baseline.

slightly stronger trend than the pentad time series from 2005 to 2019, indicating the impact of sampling changes associated with the mapping approach.

The upper 2000 m of the world's oceans have warmed on average by 5.5 ± 0.3 ZJ $yr^{-1}$ (1 ZJ = $10^{21}$ Joules) during 1958–2022 (IAP/CAS) and by 5.3 ± 0.4 ZJ $yr^{-1}$ during 1958–2020 (NCEI/NOAA pentad estimate). The 95% confidence levels are calculated using the approach of Cheng et al. (2022b). Regardless of which dataset is used, there has been a three- to four-fold increase in the rate of increase in OHC since the late 1980s. For example, according to the IAP analysis, the OHC trend for 1958–85 is 2.3 ± 0.5 ZJ $yr^{-1}$, and since 1986 the OHC trend is 8.7 ± 0.5 ZJ $yr^{-1}$.

Long-term trends are the best metrics to quantify climate changes. Short-term (e.g., annual) changes are less important but still insightful. It takes around four years of measurements for the long-term signal to emerge from short-term noise in the global ocean (Cheng et al., 2017b). A prime example of this type of internal variability is the ENSO cycle, which occurs with certain regularity in the Pacific Ocean (Cheng et al., 2019). During a prolonged La Niña event, such as the one witnessed most recently, the tropics cool overall and emits less thermal radiation to space, thereby increasing Earth's net energy imbalance and OHC (Cheng et al., 2019). Despite these internal processes, global OHC has increased steadily, regardless of the status of ENSO, owing to anthropogenic influences. When considered on an annual basis, 2022 is the hottest year ever recorded in the world's oceans. Its OHC exceeds that of 2021 by 10.9 ± 8.3 ZJ according to IAP/CAS data, and by 9.1 ± 8.7 ZJ according to NCEI/NOAA data (for the 0–2000 m water depth) (95% confidence

**Table 1**. Ranked order of the five hottest years of the world's oceans since 1955. The OHC values are anomalies for the upper 2000 m in units of ZJ relative to the 1981–2010 average.

| Rank | Year | IAP/CAS | NCEI/NOAA |
|---|---|---|---|
| 1 | 2022 | 245 | 238 |
| 2 | 2021 | 234 | 229 |
| 3 | 2020 | 221 | 211 |
| 4 | 2019 | 214 | 210 |
| 5 | 2017 | 202 | 189 |

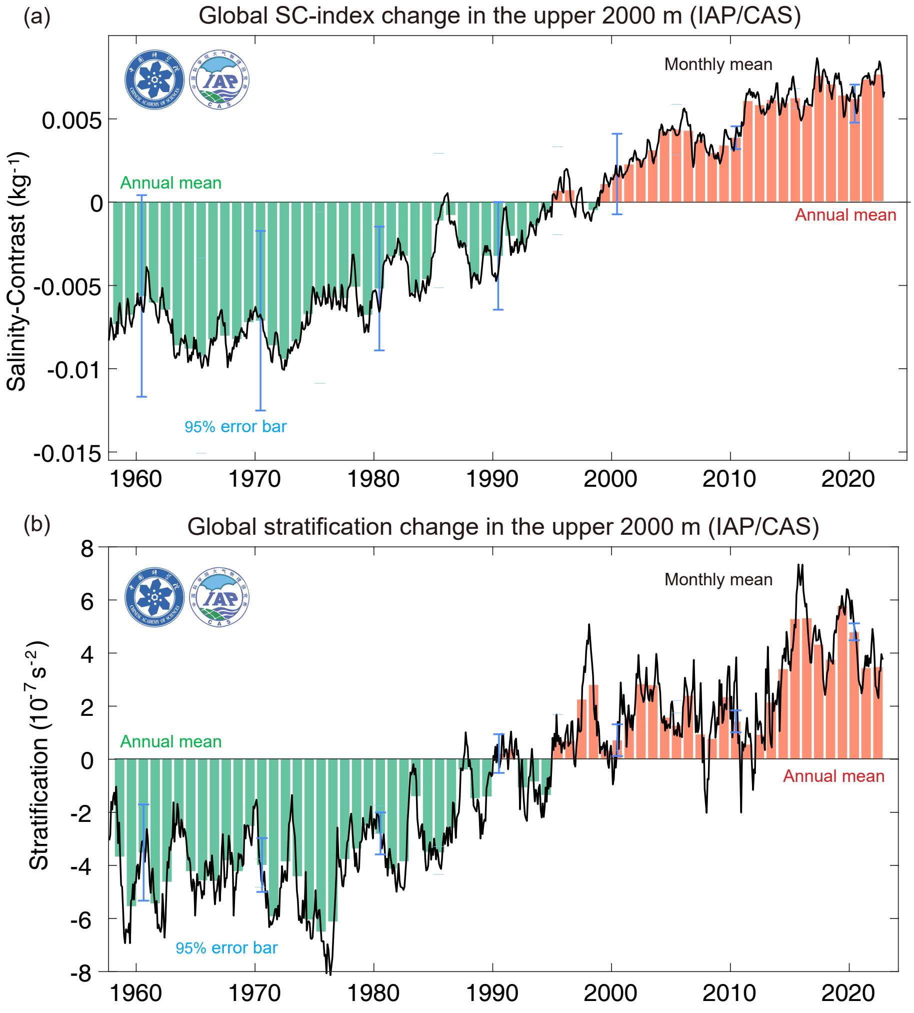

**Fig. 2.** Monthly (line) and annual (bars) changes in (a) SC index and (b) stratification in the upper 2000 m of the global ocean from 1958 to September 2022 [data updated from Cheng et al. (2017a)].

interval is presented).

Slight differences between the IAP/CAS and NCEI/NOAA estimates reflect differences in the analysis methods (mapping, quality control, data coverage, etc.), but both show excellent agreement, as indicated by the values of OHC listed in Table 1. The table lists the five hottest years ever recorded in the world's oceans. The progressive warming of the oceans is particularly notable given the contemporaneous occurrence of events thought to be exerting a strong cooling, such as the 2019–20 Australian wildfires (Fasullo et al., 2021).

Substantial changes are also seen in other oceanic metrics. Figure 2 presents the upper 2000 m SC index and stratification time series since 1959. There is a progressive increase in SC index in the past half century, while the stratification index is noisier and shows strong interannual to decadal variability in addition to an upward trend. The robust increase in SC index indicates an amplification of the 0–2000 m salinity pattern (Cheng et al., 2020). Despite the robust long-term trend, the uncertainty in salinity observations is substantial because of fewer measurements than temperature and the "salinity drift" bias in Argo data. Indeed, starting in 2015, an increased number of Sea-Bird conductivity cells used on Argo floats have salt-drifted (https://argo.ucsd.edu/data/data-faq/#sbepsal).

The increase in SC index has been robustly attributed to human influence, and this anthropogenic signal has exceeded the natural background variability (Cheng et al., 2020). While the general behavior of OHC and SC index is similar, there are some differences. For example, the SC index shows periods with little change [the 2000s, roughly—the period of the so-called global warming "hiatus" (Trenberth and Fasullo, 2013)] within an otherwise unmistakable long-term trend. In 2022, the SC index reaches 0.0.0076 ± 0.003 g kg$^{-1}$, which is the highest it has been since the late 1950s. This ocean-based metric is generally consistent with many atmosphere-based estimates and strengthens the evidence that the global water cycle has been intensified with global warming. For instance, "the fresh gets fresher and salty gets saltier in much of the ocean" pattern of change indicates the "wet gets wetter and dry gets drier" paradigm, which describes the amplification of the water cycle driven by global warming. On land, it means stronger and longer dry spells and more heavy rainfall events with potential for flooding, as has been observed (Fischer et al., 2021).

There is a punctuated increase in stratification in the past half century with large interannual variability (Fig. 2b). This increase suggests the vertical structures of the world's oceans have become more stably stratified because of changes in both temperature and salinity (Li et al., 2020a). As water layers become more stable, vertical mixing of heat, carbon, nutrients, and other concentrations is expected to decrease. Li et al. (2020a) estimated that the stratification of the ocean's upper 2000 m has increased by 5.3% since 1960 (the global 0–2000 m mean stratification, quantified by $N^2$, is 0.707 × 10$^{-5}$ s$^{-2}$), and by as much as 10%–20% in the upper 150 m. These changes are mostly related to faster warming of the surface waters compared with deeper layers, which decreases the surface water density. In 2022, the upper 2000 m stratification increases to 3.50 ± 0.37 × 10$^{-7}$ s$^{-2}$, which is amongst the top seven annual values recorded since the late 1950s. The substantial interannual fluctuations in stratification are dominated by ENSO; specifically, thermocline variations and the formation of barrier layers linked with this phenomenon (Li et al., 2020a). The La Niña condition in 2022 is associated with a vertical heat redistribution: more heat in the subsurface (100–300 m) and less heat in the near surface (0-100 m), and thus the vertical stratification is smaller than that during El Niño events.

## 4. Regional patterns of ocean warming and salinity

Spatial maps of the 2022 OHC anomaly relative to the mean 1981–2010 conditions (Fig. 3a) reveal most of the ocean areas warming significantly, while some areas (much of the Atlantic and southern oceans) are heating at a faster rate than other ocean basins. There is also a notable minimum in the northern Atlantic Ocean, consistent with the "cold blob" near the sea surface (see Rahmstorf et al., 2015). The drivers of the long-term OHC trend patterns were reviewed in Cheng et al. (2022a). There are increasing occurrences of record-shattering heatwaves and droughts in the Northern Hemisphere (Fischer et al., 2021), consistent with intensive ocean warming in the midlatitude Pacific and Atlantic oceans (Fig. 3a).

The OHC difference between 2022 and 2021 is presented in Fig. 3b and Fig. 3c for the IAP/CAS and NCEI/NOAA analyses, respectively. The two estimates show consistent large-scale patterns but the NCEI/NOAA data are noisier owing to the adopted mapping approach. In the tropical Pacific, warming anomalies around the equator and cooling anomalies on both sides of the equator in 2022 compared to 2021 (Figs. 3b and c) indicate that heat has increased in equatorial regions over the past year, partly through advection in the ocean but also through anomalous surface exchanges. Previous studies suggest that positive OHC anomalies in the western Pacific Ocean (within 5°S–5°N and 120°E–80°W) are a precursor of El Niño (McPhaden, 2012). Thus, such positive OHC anomalies and heat recharge in the equatorial region seem to forebode an El Niño condition for the following year.

The 2022 salinity anomalies relative to a 1981–2010 baseline (Fig. 4a) reveal that most of the Pacific and East Indian oceans, which are already relatively fresh climatologically, are currently undergoing a freshening, while relatively saline areas such as the midlatitude Atlantic, the Mediterranean Sea and West Indian oceans are becoming more saline. A colloquial way of stating this is "fresh areas are becoming fresher; salty areas are becoming saltier", driven by long-term atmospheric water cycle changes related espe-

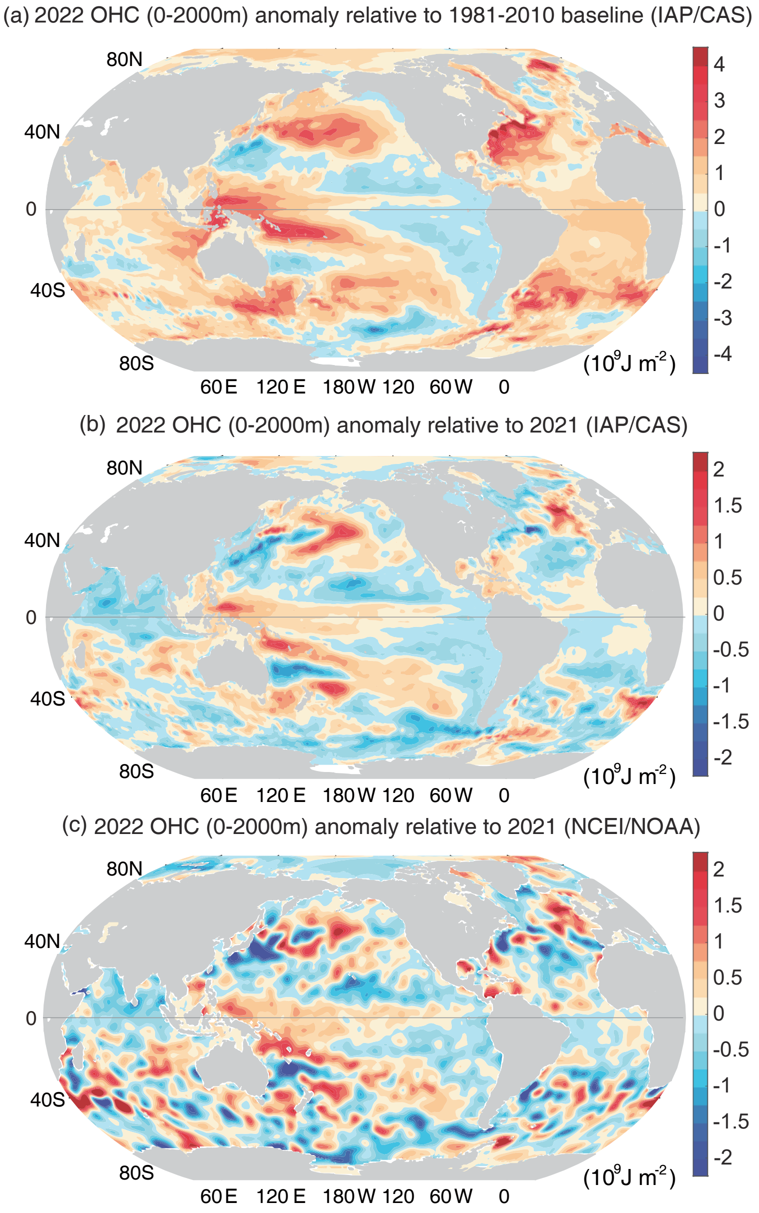

**Fig. 3.** (a) The annual OHC anomaly in 2022 relative to a 1981–2010 baseline for IAP/CAS data. (b) The difference in annual mean OHC in the upper 2000 m between 2022 and 2021. (c) As in (b) but for the NCEI/NOAA estimate. Unit: $10^9$ J m$^{-2}$. [Data updated from Cheng et al. (2017a) in (a, b) and from Levitus et al. (2012) in (c)].

cially to precipitation anomalies (Rhein et al., 2013; Zika et al., 2018; Cheng et al., 2020). The reason is that precipitation patterns are strongly tied to dynamical structures, such as convergence zones and monsoons, so that rainy areas are not random. Over oceans, where moisture is not limited, wet areas are getting wetter, and dry areas are becoming drier.

The change in salinity of the tropical Pacific Ocean in 2022 compared with 2021 (Fig. 4b) mainly reveals the impact of La Niña, and on its own should therefore not be interpreted as a trend. The upward branch of the Walker circulation over the Maritime Continent is much stronger than average, resulting in more rainfall and negative salinity anomalies but less precipitation in the west-central Pacific and south side of the equatorial Pacific where positive salinity anomalies extend into middle latitudes in the northern and southern Pacific (Fig. 4b). The latter salinity anomalies are associated with the shift in the South Pacific Convergence Zone and associated activity and precipitation.

## 5. Basin-wide OHC changes and regional hotspots

With the above spatial maps as a background, it is now possible to discuss each of the basins in detail (Fig. 5). Fur-

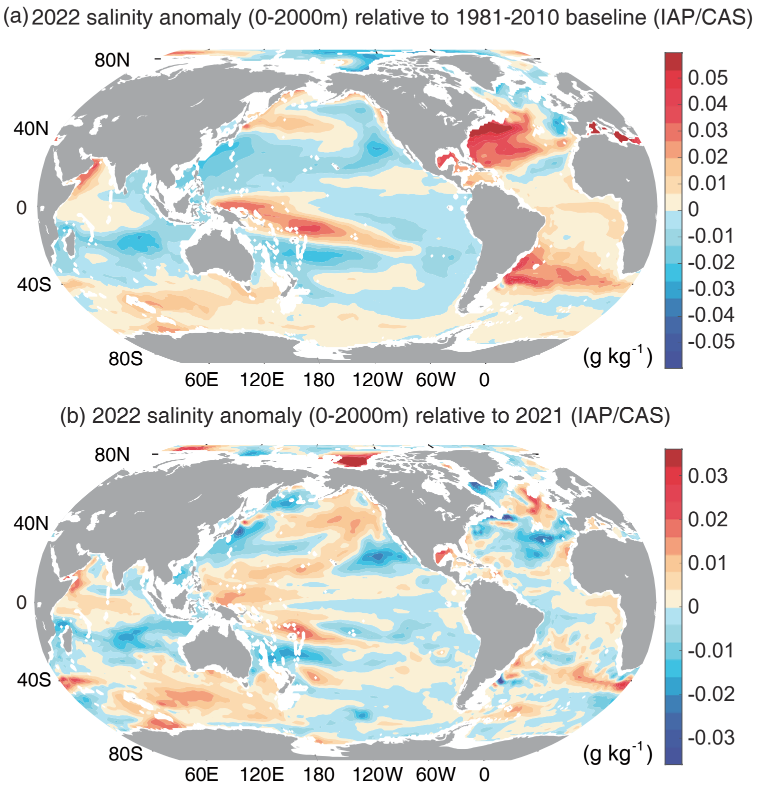

**Fig. 4.** (a) The upper 2000 m salinity anomaly in 2022 relative to a 1981–2010 baseline. (b) The difference in salinity in the upper 2000 m between 2022 and 2021 [data updated from Cheng et al. (2020)].

thermore, the figure includes the temporal evolution of OHC in each area from 1958 to 2022.

This northwest region of the Pacific Ocean, bounded by ~10°S–30°N and ~110°–170°E, is dominated by substantial interannual and decadal internal variability, especially from the Interdecadal Pacific Variability and ENSO. Since 2000, the upper 2000 m OHC values are collectively higher than in the period 1958–2000. In 2022, this Northwest Pacific OHC reaches its third highest level on record (Fig. 5a; 0.81 GJ m$^{-2}$, where 1 GJ = $10^9$ J).

The Indian OHC shows a rapid warming trend only since 2000. In 2022, the OHC in the Indian Ocean is among the top six years ever recorded (Fig. 5b; 0.63 GJ m$^{-2}$). The southeast Indian Ocean stands out, with regionally enhanced warming around 2000 (Fig. 3a), probably linked to the increased heat transport from the Pacific, as was the case for the 2010–2012 multi-year La Niña (Feng et al., 2015; Li et al., 2017). The warming in this region is expected to drive extreme heatwave events that leave devastating consequences on coral ecosystems (Wernberg et al., 2013). The decrease in OHC from 2020 to 2022 is consistent with the negative Indian OHC tendency during La Niña (Cheng et al., 2019), driven mainly by decreasing the heat transport of the Indonesian Throughflow during the decaying stage of La Niña (Li et al., 2020b; Volkov et al., 2020).

The tropical Atlantic Ocean (10°–30°N), a region important for hurricane development (Trenberth et al., 2018), shows a continual increase in OHC since the late 1950s (Fig. 5c). In 2022, the upper 2000 m OHC ranks second in this region (0.77 GJ m$^{-2}$), slightly lower than the 2016 level (0.78 GJ m$^{-2}$) . The North Atlantic Ocean has warmed continuously from the late 1950s until around the year 2000, and the 2022 OHC reaches a record level (1.06 GJ m$^{-2}$). The value is almost identical to the 2021 level (Fig. 5e). Part of the warming derives from reduced aerosol concentrations (Murakami, 2022), and the result has been substantial increases in tropical cyclone and hurricane activity in the Atlantic (Vecchi et al., 2021; Truchelut et al., 2022).

The Mediterranean Sea OHC in 2022 is identical to the 2021 level (1.35 GJ m$^{-2}$) (Fig. 5d), and these record-high OHCs are consistent with the widespread and prolonged marine heat waves recorded in recent years in its central and western parts. However, an independent ocean reanalysis data (CMS-MEDREA) updating to November 2022 indicates a reduced OHC in 2022 than 2021, probably associated with the different methodology and relative uncertainty in generating observations-only estimates and reanalyses (Simoncelli et al., 2022).

In the North Pacific Ocean (30°–62°N) and South Pacific (not shown), a large-scale warming condition and persistent marine heat waves [referred to as "The Blob" (Scannell et al., 2020)] are evident in recent years (Figs. 3 and 5), in part consistent with La Niña teleconnections. In 2022, the upper 2000 m OHC reaches a record level (0.95 GJ m$^{-2}$) by a large margin, which supports the extreme events witnessed, such as intensive heat waves and deoxygenation,

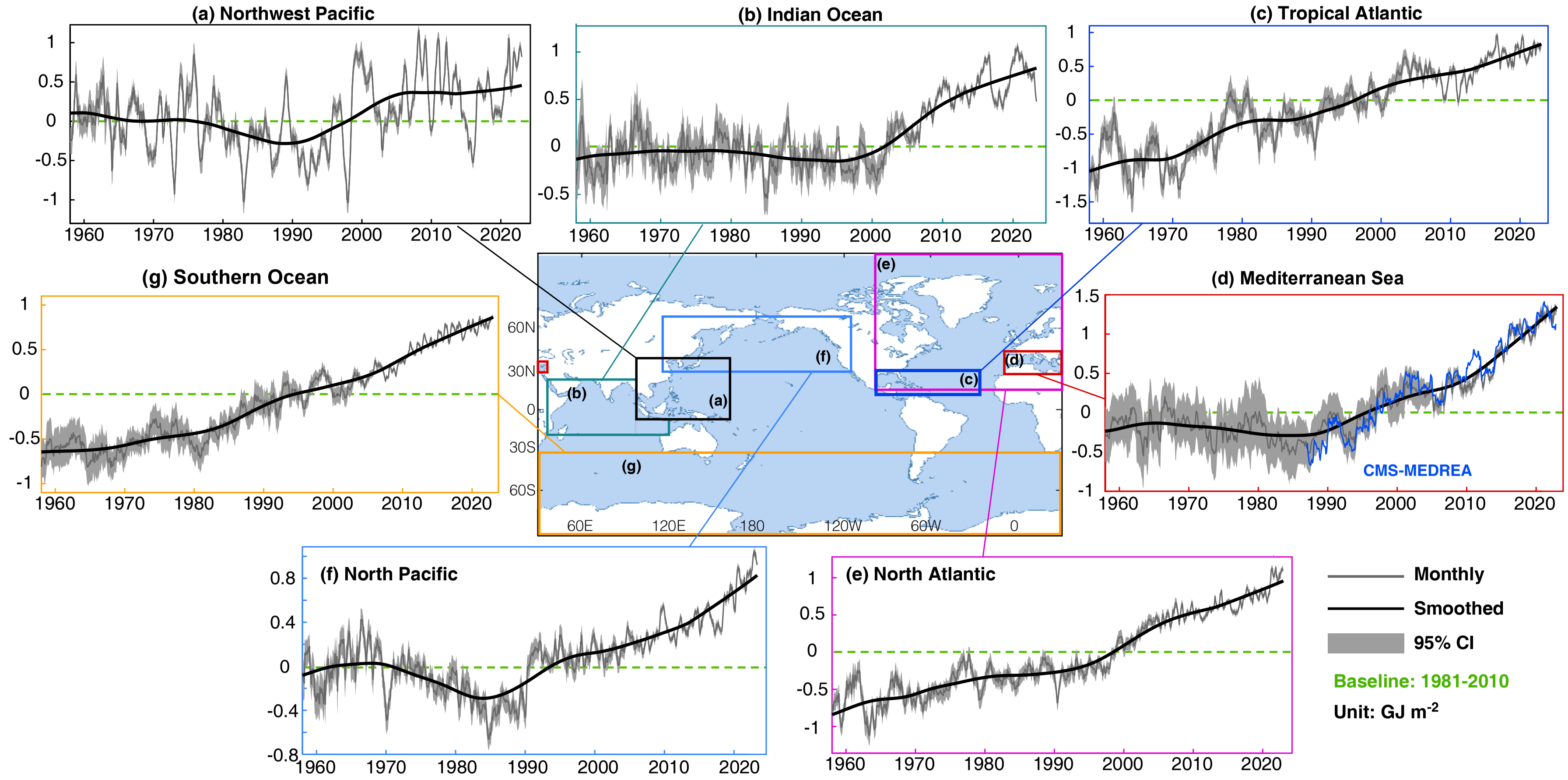

**Fig. 5.** Regional observed upper 2000 m OHC change from 1958 through 2022 relative to a 1981–2010 baseline. The time series (black lines) are smoothed by LOWESS (locally weighted scatterplot smoothing) with a span width of 240 months. The gray shaded areas are the 95% confidence intervals. [Data updated from Cheng et al. (2017a)].

and poses a substantial risk to marine life in this region.

The Southern Ocean has experienced a long-term warming trend since the 1950s (Fig. 5g) and has been the major heat and carbon sink for the past century. The OHC level in 2022 for this region (0.82 GJ $m^{-2}$) exceeds last year's record (0.81 GJ $m^{-2}$), albeit with uncertainty. The interannual change in OHC in the southern oceans is also linked to ENSO (e.g., Ding et al., 2011; Wang et al., 2022). The recent La Niña in the tropical Pacific deepened the Amundsen Sea Low through a Rossby wave train (e.g., Li et al., 2021), which favored an enhanced heat uptake via the upwelled deep water and heat convergence on the northern flank of the Antarctic Circumpolar Current (Armour et al., 2016; Wang et al., 2022).

## 6. Persistence of decadal OHC patterns

As the global Argo network has now collected data for an unprecedented period of time (almost two decades), it is worthwhile exploring the persistence of the decadal OHC patterns. Figure 6 shows the upper 2000 m OHC anomalies for the periods 2003–12 and 2013–22 relative to the global mean. In both decades, OHC shows a robust increase in the Indo-Pacific region, the Kuroshio extension, the West and South Pacific, and the midlatitude Atlantic, in both hemispheres. Cooling trends persist in the Southwest Indian and eastern Pacific oceans. The latter highlights the dominance of La Niña and negative Pacific Decadal Oscillation/Interdecadal Pacific Variability in both periods. Nevertheless, most of these signals are consistent with the response of OHC to anthropogenic forcing (Fasullo et al., 2018) and long-term OHC changes from 1958 to 2020 (Cheng et al., 2022a). The patterns show a strong degree of correlation ($r$ = 0.45), particularly when considered outside of the North Atlantic Ocean ($r$ = 0.60). Despite being limited in duration to two decades, the observed anomalies support the idea of a persistent pattern of change across the Pacific Ocean consistent with a strengthening of the Walker circulation, with impli-

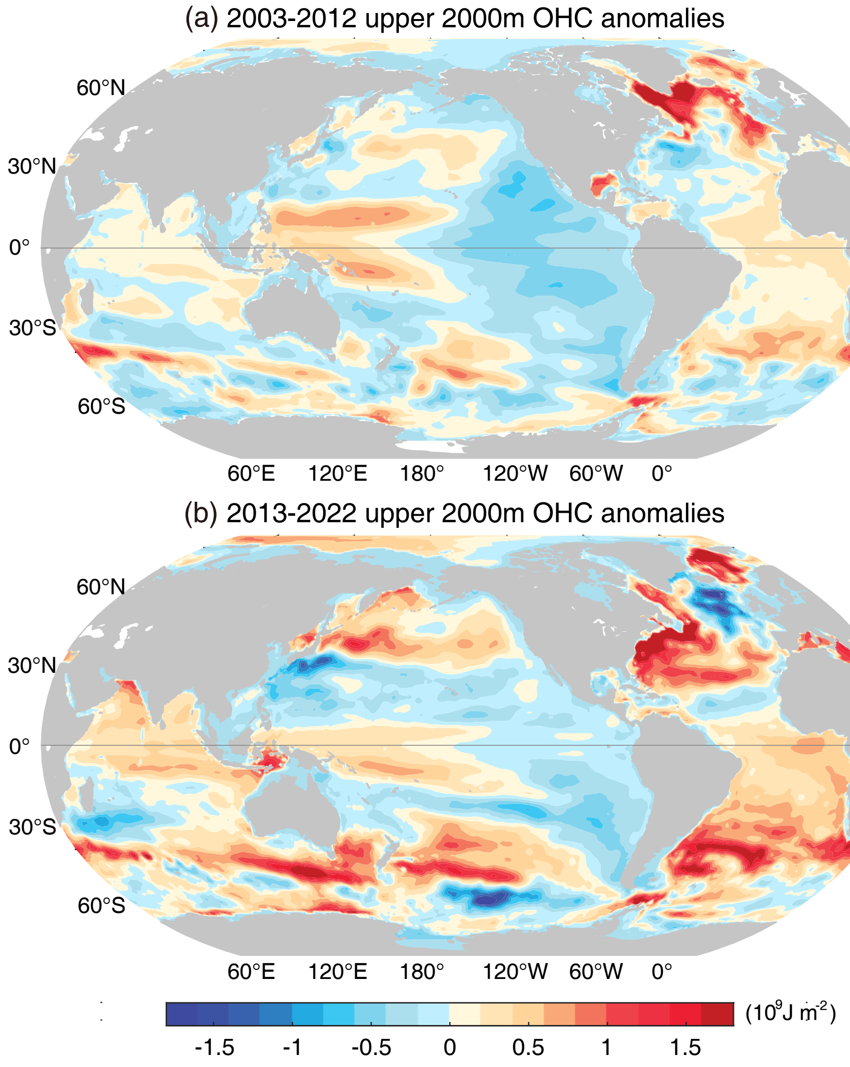

**Fig. 6.** Anomalies of upper 2000 m OHC from (a) 2003 to 2012 and (b) 2013 to 2022, relative to a 1981–2010 baseline but with the global mean value removed for each map [data updated from Cheng et al. (2017a)].

cations for a range of key topics including the impacts of sea level rise, climate sensitivity, and the terrestrial hydroclimate (Fasullo and Nerem, 2018; Nguyen et al., 2021).

## 7. Concluding remarks

Based on analyses from two international groups (IAP/CAS and NCEI/NOAA), this paper provides estimates of the OHC, salinity, and stratification in 2022. While these groups use different approaches to form a continuous record of temperature change, the results are consistent and mutually reinforcing.

First, we find that the oceans are continuing to warm globally, with yet another new 0–2000 m OHC record reached in 2022. The inexorable climb in ocean temperatures is the inevitable outcome of Earth's energy imbalance, primarily associated with increasing concentrations of greenhouse gases. The global long-term warming trend is so steady and robust that annual records continue to be set with each new year. The warming has accelerated in recent decades, with a faster rate of warming evident since roughly 1990 (Cheng et al., 2022a, b). Similarly, the SC index has increased, signifying more extreme salinity anomalies and an imprint of global water cycle amplification on the upper ocean. We also show a sustained increase in ocean stratification, with ocean waters becoming increasingly more stable over time, although with more variability than other fields.

Next, regional warming patterns are displayed and local hot and cold spots identified. All basins have experienced some warming with internal variability superposed atop the long-term trends. In 2022, four out of seven regions reached record levels of heat content for their upper 2000 m OHC, and all seven regions were among the top ten warmest years.

In addition to the ocean metrics presented in this study, other changes indicating ocean health that can be included in the future include ocean oxygen change and pH. Furthermore, a recent study (Ren et al., 2022) proposed another metric to synthesize changes in ocean temperature and salinity: the spatial inhomogeneity. Under greenhouse warming, oceans are becoming less homogeneous in terms of thermohaline properties, with the thermohaline inhomogeneity index [defined as the spatial standard deviation of seawater in density-spicity space (Huang et al., 2018)] having increased by ~2.4% compared to the 1960 level.

***Acknowledgements***. The IAP/CAS analysis is supported by the National Natural Science Foundation of China (Grant Nos. 42122046 and 42076202) and the Strategic Priority Research Program of the Chinese Academy of Sciences (Grant No. XDB42040402). NCAR is sponsored by the US National Science Foundation. The efforts of Dr. FASULLO in this work were supported by NASA Awards 80NSSC17K0565 and 80NSSC22K0046, and by the Regional and Global Model Analysis (RGMA) component of the Earth and Environmental System Modeling Program of the U.S. Department of Energy's Office of Biological & Environmental Research (BER) via National Science Foundation IA 1947282. The efforts of Dr. A. MISHONOV were supported by NOAA (Grant No. NA19NES4320002 to CISESS-MD at the University of Maryland). The IAP/CAS data are available at http://www.ocean.iap.ac.cn/ and https://msdc.qdio.ac.cn/. The NCEI/NOAA data are available at https://www.ncei.noaa.gov/products/climate-data-records/global-ocean-heat-content. This study has been conducted using also E.U. Copernicus Marine Service Information (https://marine.copernicus.eu/) for the Mediterranean OHC estimate. G. Li is supported by the Young Talent Support Project of Guangzhou Association for Science and Technology.

***Open Access*** This article is licensed under a Creative Commons Attribution 4.0 International License, which permits use, sharing, adaptation, distribution and reproduction in any medium or format, as long as appropriate credit is given to the original author(s) and the source, plus a link to the Creative Commons license, and indications of any changes made. The images or other third-party material in this article are included in the article's Creative Commons license, unless indicated otherwise in a credit line to the material. If material is not included in the article's Creative Commons license and intended use is not permitted by statutory regulation or exceeds the permitted use, the user will need to obtain permission directly from the copyright holder. To view a copy of this license, visit http://creativecommons.org/licenses/by/4.0/.
